# Examining the role of olfaction in dietary choice

**DOI:** 10.1016/j.celrep.2021.108755

**Published:** 2021-02-16

**Authors:** Montana H. Boone, Jing Liang-Guallpa, Michael J. Krashes

**Affiliations:** 1 Diabetes, Endocrinology, and Obesity Branch, National Institute of Diabetes and Digestive and Kidney Diseases (NIDDK), National Institutes of Health, Bethesda, MD 20892, USA; 2 National Institute on Drug Abuse (NIDA), National Institutes of Health, Baltimore, MD 21224, USA; 3 NIH-Brown University Graduate Program in Neuroscience, Bethesda, MD 20892, USA; 4 Lead contact

## Abstract

Obesity is frequently caused by calorie-rich dietary choices across the animal kingdom. As prandial preference toward a high-fat diet develops in mice, an anti-preference or devaluation of a nutritionally balanced but less palatable standard chow diet occurs concomitantly. Although mechanistic insights underlying devaluation have been observed physiologically in the brain, it is unclear how peripheral sensory processing affects food choice. Because olfactory cues and odor perception help coordinate food preference and intake, we determine the role of smell in the targeted consumption of a high-fat diet and simultaneous devaluation of a standard chow diet. Using inaccessible food and loss-of-function manipulations, we find that olfactory information is neither sufficient nor necessary for both the acute and chronic selection of high-fat diet and coincident diminished value of standard diet. This work suggests alternative means are behind the immediate and sustained consumption of high-fat diet and concurrent standard diet devaluation.

## In Brief

Olfaction is considered integral to food choice and preference formation. Boone et al. use a behavioral approach to determine the salience of olfactory cues in diet selection. They find that olfaction is neither sufficient nor required for both high-fat-diet preference and standard diet devaluation.

## Graphical Abstract

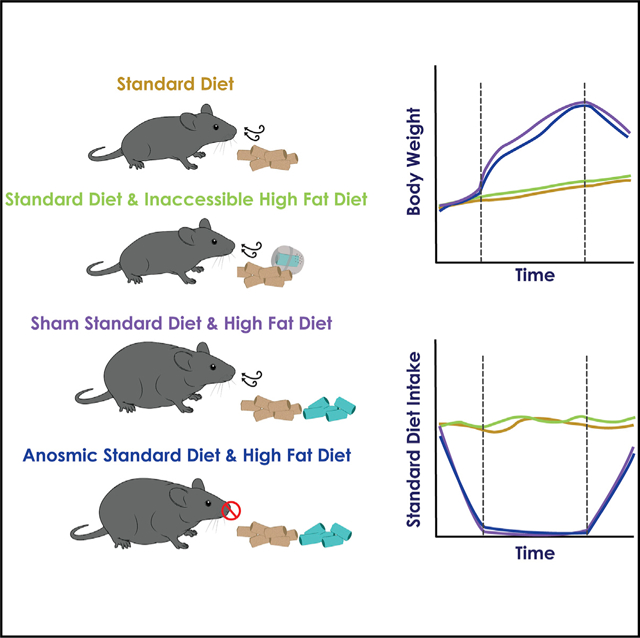

## INTRODUCTION

As the prevalence of obesogenic foods increased in the past several decades, obesity rates skyrocketed (Ferrario et al., 2016). Over 40% of adults in the United States alone have obesity, correlating with increased risk of cardiovascular disease and other comorbidities. Obesity is strongly associated with preferential consumption of diets high in fat (Guo et al., 2009; Hamilton, 1964; Briggs et al., 2013). Although high-fat-diet (HFD) intake can endanger lives, humans consciously hold this preference, ranking food with higher fat content as more palatable (Drewnowski et al., 1985). Interventions for diet-induced obesity are minimally or temporarily successful as healthier food options are devalued even after short-term HFD exposure (Hamilton, 1964). Treatment is additionally confounded by the physiological and motivational changes outlasting high fat ingestion (Bachmanov et al., 2001; Mazzone et al., 2020; Beutler et al., 2020; Lippert et al., 2020; Vogt et al., 2014). HFD-induced obese mice show selective changes in neural response of both hypothalamic and mesolimbic brain regions to a devalued standard diet (SD), partly due to an attenuated hypothalamic response to calories or lipids (Beutler et al., 2020; Mazzone et al., 2020). Although post-ingestive properties contribute to food choice, an important unanswered question is whether sensory modalities play a role in HFD-induced devaluation of a nutritionally balanced but less palatable SD.

Because nutrients and fats in food are registered by chemosensation before any other sensory modality (Julliard et al., 2017; Boesveldt and Lundström, 2014), smell, texture, and taste are vital for appetite regulation (Soria-Gómez et al., 2014) and HFD preference formation (Sørensen et al., 2003). Real-time recordings of population dynamics from orexigenic agouti-related peptide (AgRP) or anorectic proopiomelanocortin (POMC) neurons reveal rapid inhibition or activation, respectively, to hidden peanut butter (Chen et al., 2015), suggesting that the smell of food quickly modulates the firing properties of key feeding regulators before nutrients are tasted or consumed. Further studies using Pavlovian conditioning demonstrate that AgRP activity is inhibited by a learned stimulus (Betley et al., 2015) or the inclusion of calories in a non-caloric substrate (Su et al., 2017). Thus, discrete food-related cues rapidly regulate AgRP neurons and affect food-seeking behavior.

Odor or gustatory perception is weakened in people with higher body-mass index (Simchen et al., 2006; Fernández-Aranda et al., 2016). In rodents, HFD consumption decreases olfaction detection or discrimination regardless of the presence of obesity (Takase et al., 2016; Thiebaud et al., 2014; Richardson et al., 2004). Further studies show that temporary and permanently induced anosmia lead to resistance against diet-induced obesity and that anosmia reduces prior obesity phenotypes, such as fat mass and fat food consumption (Riera et al., 2017; Kinney and Antill, 1996). However, this reduction in HFD intake may stem from methodological approaches to elicit anosmia. Regeneration of olfactory neurons occurs rapidly following either bilateral sectioning of the olfactory nerve (Harding et al., 1977) or inducible toxin-based strategies (Kang et al., 2015), making it difficult to discern the relative levels of residual smelling capacity. Moreover, as acute anosmia has been shown to engender states of depression and stress (van Riezen et al., 1977; Song and Leonard, 2005; Cairncross et al., 1977), repeated olfactory sensory neuron ablation may obscure causation of HFD-induced obesity protection.

Because olfaction has been shown to play a critical, yet controversial role in HFD preference and maintenance, we use a food choice/accessibility paradigm in rodents to assess either the sufficiency or necessity of olfaction in both HFD preference and SD devaluation. Here, we find that the smell of HFD alone is ineffective in altering the valuation and consumption of SD or subsequent intake of HFD. In support of this notion, we show that olfaction is entirely dispensable for short- and long-term HFD preference and corresponding SD devaluation. Anosmia did not protect against diet-induced obesity or affect feeding preference patterns. Therefore, sensory detection of calories through olfaction is not necessary for food selection and consumption in mice.

## RESULTS

### Olfactory perception of HFD without consumption does not alter home-cage food intake or induce SD devaluation

**Figure 1.**
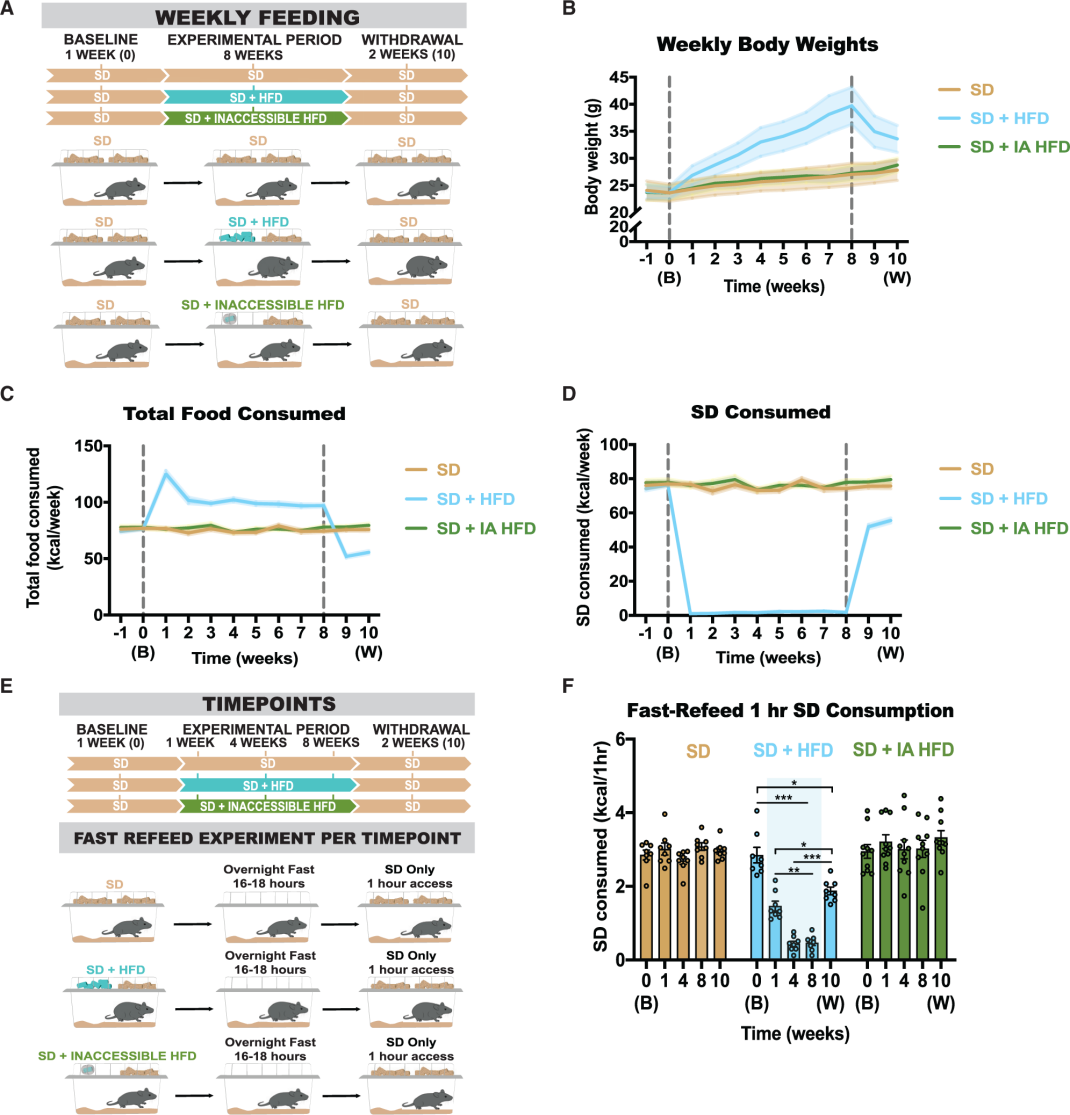
Devaluation of SD occurs only when HFD is accessible for consumption (A) Experimental timeline and group schematic for home-cage measurements. (B–D) Weekly measurements of (B) weight (repeated measures [RM] two-way ANOVA, week × group: F (22, 253) = 17.10, p < 0.0001); (C) total number of calories consumed (RM two-way ANOVA, week × group: F (22, 253) = 55.85, p < 0.0001); and (D) number of SD calories consumed (RM two-way ANOVA, week × group: F (22, 253) = 145.9, p < 0.0001). (E) Experimental timeline and group schematic for fast-refeed test with 1-h SD access. (F) Within-subject comparison of 1-h SD fast-refeed consumption across testing sessions (RM two-way ANOVA, time × group: F (8, 88) = 23.91, p < 0.0001, Tukey’s multiple comparisons). Dotted lines in (B)–(D) delineate window of HFD availability or inaccessible HFD. B, baseline; W, withdrawal. Shaded blue area in (F) represents HFD home-cage availability. All groups had a mix of males and females. n = 8–9 per group. All error bars and shaded regions of (B)–(D) represent mean ± SEM. *p < 0.05, **p < 0.01, ***p < 0.001.

To examine whether olfactory detection of HFD alone can influence consumption of SD, we enclosed HFD in a porous, opaque tea ball so that mice could smell the HFD without the ability to consume it. Mixed-sex adult mice were given SD for 1 week before being divided equally among 3 groups, as follows: 1 group continued on SD only, 1 group was given SD + HFD for an 8-week experimental period followed by SD only during a 2-week withdrawal phase, and a final group was given SD and inaccessible HFD for 8 weeks and SD only during the withdrawal phase (Figure 1A). Mice given a dietary choice between HFD and SD show significant weight gain, eat a greater number of calories in total, and show selective decrease in SD intake (Figures 1B and 1D). Although mice show varied body mass changes after long-term HFD consumption (Yang et al., 2014), the reduction of SD occurs in all mice and is not correlated with amount of body mass accrual (Figures S1A–S1C). Following a withdrawal period, HFD-fed mice lost substantial body weight due to inadequate consumption of calories (Guo et al., 2009; Mazzone et al., 2020; Figures 1B–1D). Similar to their littermate SD controls, mice with inaccessible HFD maintain stable body weights and SD consumption levels regardless of HFD odor (Figures 1B–1D).

To confirm that the smell of HFD does not affect post-fasting consumption of SD, mice were food deprived and longitudinally tested for 1 h of SD intake at baseline, 1, 4, and 8 weeks into the experimental period and 2 weeks after withdrawal from HFD/inaccessible HFD (Figure 1E). As expected, mice challenged with HFD exhibit a significant reduction or passive devaluation of SD even during periods of physiological hunger; this reduction in post-fasting consumption for HFD-fed mice partly remains after withdrawal from HFD (Figure 1F). In contrast, mice with home-cage access to SD with or without inaccessible HFD exhibit no changes in SD consumption across all time points (Figure 1F), demonstrating that the home-cage experience of smelling the HFD alone is insufficient to devalue SD in hungry animals.

Similar to some drugs of abuse, reintroduction of highly palatable food after a withdrawal period results in a relapse phase. To assess whether exposure to the smell of HFD exacerbates intake of HFD similarly to animals with prior experience consuming HFD, we divided mice into the same 3 groups over the course of 3 weeks, comprising a 1-week baseline, 1-week experimental phase, and a 1-week withdrawal period (Figure S2A). As observed above, mice maintained on SD with or without inaccessible HFD display comparable body weights and SD consumption (Figures S2B–S2D). After the withdrawal period, all mice were food deprived overnight, and then a 1-h fast-refeed test was performed in the presence of both SD and HFD (Figure S2A). All groups of mice show a strong preference for HFD calories, but this was only elevated in mice with prior experience consuming HFD (Figure S2E), implicating HFD olfactory familiarity in the absence of ingestion fails to intensify HFD intake.

As initial exposure to HFD results in early consumption and preference for HFD (Figure S2E), we hypothesized that the initial exposure to HFD smell alone could affect acute SD intake in physiologically hungry mice. Mice with home-cage access to SD were calorically restricted overnight and then tested for 1-h SD intake the following morning in the presence of inaccessible HFD or inaccessible SD (Figure S2F). SD intake is comparable between conditions, suggesting HFD odor alone is unable to impact fast-refeed SD intake (Figure S2G).

### Prior HFD exposure does not enhance salience of HFD olfactory cues

**Figure 2.**
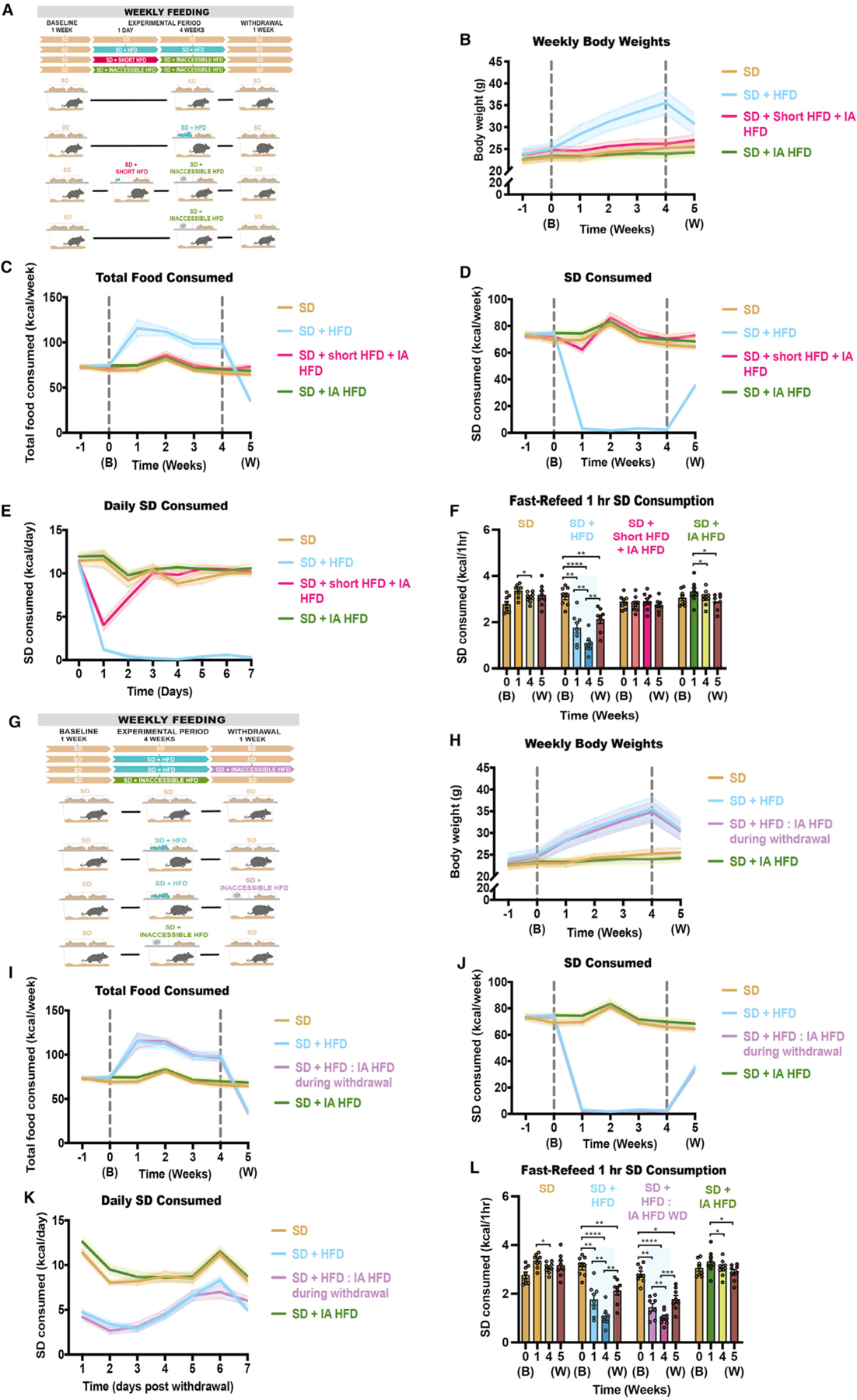
Long-term accessibility, not just exposure, of HFD is necessary for continued devaluation of SD (A) Experimental paradigm and group schematic for home-cage measurements in mice undergoing various inaccessible exposure times to HFD. (B–D) Weekly measurements of (B) weights (RM two-way ANOVA, week × group: F (18, 168) = 17.70, p < 0.0001); (C) total number of calories consumed (RM two-way ANOVA, week × group: F (18, 168) = 27.79, p < 0.0001); and (D) number of calories consumed in SD only (RM two-way ANOVA, week × group: F (18, 168) = 251.3, p < 0.0001). (E) Daily measurements of the number of calories consumed in SD post-withdrawal (RM two-way ANOVA, week × group: F (18, 168) = 10.00, p < 0.0001). (F) Number of SD calories consumed during a 1-h fast-refeed session at baseline, at 1 and 4 weeks post-HFD exposure/baseline, and at 5 weeks after a 1-week withdrawal from HFD (RM two-way ANOVA, week × group: F (9, 84) = 22.22, p < 0.0001). (G) Experimental paradigm and group schematic for home-cage measurements in mice undergoing various accessible exposure times to HFD. (H–J) Weekly measurements of (H) weights (RM two-way ANOVA, week × group: F (18, 168) = 19.77, p < 0.0001); (I) total number of calories consumed (RM two-way ANOVA, week × group: F (18, 168) = 33.62, p < 0.0001); and (J) number of calories consumed in SD only (RM two-way ANOVA, week × group: F (18, 168) = 163.5, p < 0.0001). (K) Daily measurements of the number of calories consumed in SD right after the baseline period ends (RM two-way ANOVA, week × group: F (21, 196) = 24.31, p < 0.0001). (L) Number of SD calories consumed during a 1-h fast-refeed session at baseline, at 1 and 4 weeks post-HFD exposure/baseline, and at 5 weeks after a 1-week withdrawal from HFD (RM two-way ANOVA, week × group: F (9, 84) = 19.80, p < 0.0001). Shaded blue area in (F) and (L) represent HFD home-cage availability. All groups had a mix of males and females. n = 8 per group. All error bars and shaded regions of (B)–(E) and (H)–(K) represent mean ± SEM. *p < 0.05, **p < 0.01, ***p < 0.001, ****p < 0.0001.

To determine how odor plays a role in palatable food intake after previous exposure to HFD, mice were first exposed to HFD before inaccessible HFD was present in the cage. Mixed-sex mice were divided equally between four groups, as follows: (1) mice given access to SD during the entire course of the experiment; (2) mice given SD + HFD during the experimental period only; (3) mice given SD, a one-time exposure to calorie-matched HFD equaling their daily caloric load, and SD + inaccessible HFD during the experimental period; and (4) mice given SD + inaccessible HFD during the experimental period only (Figure 2A). Like previous results, only mice given prolonged HFD access drastically increase body weights and decrease SD consumption, whereas mice given inaccessible HFD show similar trends to SD controls (Figures 2B–2D). Mice given a short exposure to HFD did not devalue the SD as much as mice exposed to HFD for the entire experimental period (Figure 2D); however, mice require a couple of days after the brief HFD exposure to return to average levels of SD consumption (Figure 2E). To confirm that olfactory cues alone are not salient during hunger, mice were food deprived and longitudinally tested for 1-h SD intake at baseline, 1 week, and 4 weeks into the experimental period and 1 week after withdrawal from HFD (Figure 2F). The amount of SD consumption by mice exposed to short-term HFD and inaccessible HFD does not differ from the amount of SD consumed by either the mice exposed only to SD or mice exposed to SD and inaccessible HFD (Figure 2F), implying that prior short-term HFD exposure does not update the salience of HFD olfactory cues.

Because short-term HFD exposure did not impact feeding patterns, we hypothesized that prior long-term HFD consumption would affect the salience of olfactory cues or subsequent SD devaluation. Mixed-sex mice were divided equally between four groups, as follows: (1) mice given access to SD during the entire course of the experiment, (2) mice given SD + HFD during the experimental period only, (3) mice given SD + HFD during the experimental period and SD + inaccessible HFD during the withdrawal period, and (4) mice given SD + inaccessible HFD during the experimental period only (Figure 2G). As expected, although both groups exposed to HFD during the experimental period show increased body weights and decreased caloric consumption of SD (Figures 2H–2J), the presence of inaccessible HFD, or olfactory cues of HFD, during the withdrawal period does not significantly alter daily rate of change in SD consumption (Figures 2I–2K). Additionally, both groups exposed to HFD during the experimental period decrease their consumption of SD during all post-baseline 1-h fast-refeeds (Figure 2L).

### Olfaction is not required for home-cage HFD preference or SD devaluation

**Figure 3.**
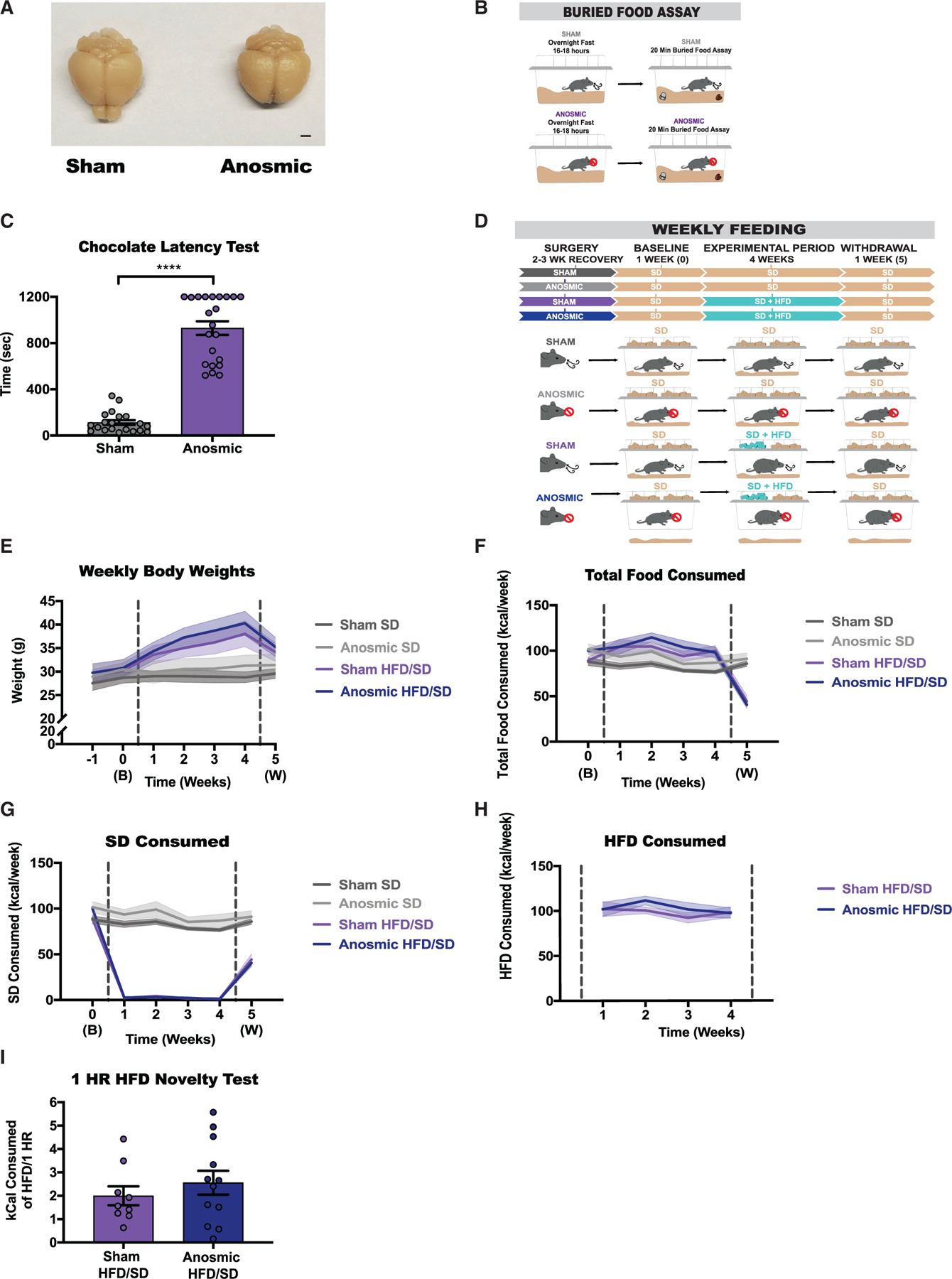
Anosmia does not protect against HFD-induced weight gain, HFD preference, or SD anti-preference (A) Representative photographs of mice that underwent either a sham or olfactory ablation surgery resulting in bilateral removal of the olfactory bulb. Scale bar represents 2 mm. (B) Experimental paradigm and group schematic for buried food assay. (C) Latencies (s) to discovering the food object (unpaired t test [two-tailed’, p < 0.0001), n = 20–22 per group. (D) Experimental timeline and group schematic for home-cage measurements. (E–I) Weekly measurements of (E) weight (RM two-way ANOVA, week × group: F (18, 228) = 12.13, p < 0.0001), n = 9–13 per group; (F) total number of calories consumed (RM two-way ANOVA, week × group: F (15, 190) = 10.87, p < 0.0001), n = 9–13 per group; (G) number of SD calories (RM two-way ANOVA, week × group: F (15, 190) = 74.59, p < 0.0001), n = 9–13 per group or (H) HFD consumed (RM two-way ANOVA, week × group: F (3, 63) = 0.6864, p = 0.5637), n = 9–13 per group; and (I) number of HFD calories consumed in sated animals during a 1-h novel exposure to HFD (unpaired t test [two-tailed], p = 0.4024), n = 9–12 per group. All groups had a mix of males and females. Dotted lines in (E)–(H) delineate window of HFD availability. All error bars and shaded regions of (E)–(H) represent mean ± SEM. ****p < 0.0001.

Because chemosensory cues serve as essential indicators for food foraging and appreciation (Sørensen et al., 2003; Julliard et al., 2017; Boesveldt and Lundström, 2014), we hypothesized that anosmia may reduce acute and/or chronic preference toward HFD consumption. Mice underwent either a sham surgery or a procedure involving the complete bilateral removal of the olfactory bulb (Figure 3A). To validate the efficiency of the bulbectomy, we performed an olfaction-dependent foraging test whereby both hungry sham and bulbectomized animals were tasked with finding a hidden chocolate chip buried under bedding (Figure 3B). Shams locate the food in less than 2 mins on average with a 0% failure rate, whereas bulbectomized mice take 15.5 mins on average with around a 40% failure rate, confirming the efficacy of the anosmia-inducing surgeries (Figure 3C).

Following a 2- to 3-week recovery period, all sham and anosmic mice were maintained on SD through a 1-week baseline period. During a 4-week experimental period, half of the mice were given access to SD + HFD and the other half remained on SD. Lastly, all mice were given SD only through a 1-week withdrawal period (Figure 3D). Both sham and anosmic mice maintained on SD exhibit comparable body weight and SD consumption throughout the experiment (Figures 3E–3G). Mice challenged with HFD display rapid weight gain, elevated total caloric intake, and a selective preference for HFD, regardless of their ability to smell (Figures 3E–3H). To determine if anosmia impacts the response to the initial exposure to HFD, all mice were given 1 h of HFD exposure in the sated condition. Consistent with long-term HFD challenge, both sham and anosmic mice show comparable initial HFD intake (Figure 3I). Critically, the reduced SD intake displayed by HFD-challenged mice during the experimental period remains even after a 1-week withdrawal phase, leading to a precipitous drop in body weight (Figures 3E–3G).

### Intact olfaction is not necessary for devaluation of SD during periods of hunger

**Figure 4.**
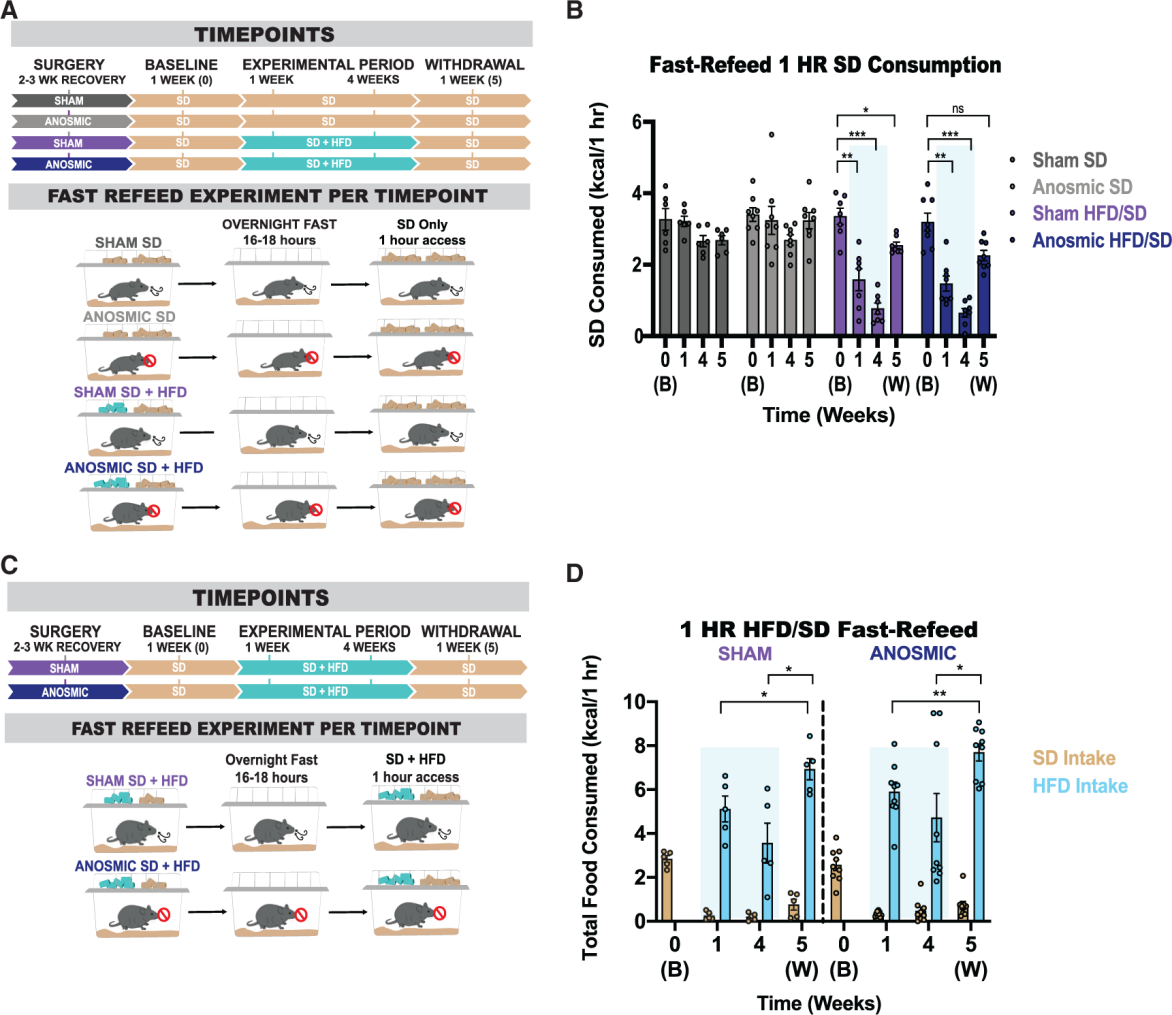
Selective SD devaluation occurs independent of physiological hunger state in both anosmic and olfaction-intact mice (A) Experimental timeline and group schematic for fast-refeed test with 1-h SD access. (B) Within-subject comparison of 1-h SD fast-refeed consumption across testing sessions (RM two-way ANOVA, time × group: F (9, 75) = 6.978, p < 0.0001, Tukey’s multiple comparisons), n = 6–8 per group. (C) Experimental timeline and group schematic for fast-refeed test with 1-h SD and HFD access. (D) Within-subject comparison of 1-h SD (RM two-way ANOVAs, time × group: F (3, 36) = 0.6966, p = 0.6966, Tukey’s multiple comparisons), n = 5–9 per group; and HFD (RM two-way ANOVAs, time × group: F (2, 24) = 0.06694, p = 0.9354, Tukey’s multiple comparisons) fast-refeed consumption across testing sessions, n = 5–9 per group. All groups had a mix of males and females. Shaded blue area in (B) and (D) represent HFD home-cage availability. All error bars represent mean ± SEM. *p < 0.05, **p < 0.01, ***p < 0.001, ****p < 0.0001.

We recently showed a rapid, durable, and persistent devaluation of SD in mice exposed to HFD (Mazzone et al., 2020). To assess whether this SD devaluation requires intact olfaction, both sham and anosmic mice were food deprived overnight and provided 1 h of SD access (Figure 4A). During baseline when all mice only had home-cage access to SD, both sham and anosmic mice consume comparable SD calories during this 1-h fast-refeed period (Figures 4A and 4B). Despite evidence that olfaction can be modulated by internal state (Hanci and Altun, 2016; Tuerdi et al., 2018), SD intake in both hungry sham and anosmic animals is similar (Figure S3A). Although 1-h SD intake is consistent between fasted sham and anosmic mice naive to HFD, home-cage HFD challenge significantly attenuates SD intake during the experimental period, an effect that persisted after home-cage HFD withdrawal (Figures 4A and 4B). In summary, olfaction is not necessary for either fasting-induced SD intake in HFD-naive mice or SD devaluation in HFD-exposed mice, and these effects are decorrelated with body mass accrual (Figures S3B–S3E).

Although we previously determined that SD devaluation in HFD-challenged animals was explicitly selective for SD calories, as hungry animals voraciously targeted and consumed HFD during a fast-refeed (Mazzone et al., 2020), the role of olfaction in this consummatory choice is unclear. To uncouple SD devaluation from demotivation to eat, we repeated the 1-h fast-refeed experiments in HFD-exposed mice in the presence of both HFD and SD (Figure 4C). Both sham and anosmic mice selectively overconsume HFD over SD (Figures 4C and 4D). Upon a 1-week withdrawal from HFD, both sham and anosmic mice overconsumed HFD during a 1-h fast-refeed period in excess of double the amount of SD calories consumed at baseline prior to the HFD experience (Figures 4C and 4D). Thus, the relapse-like overeating of HFD mice observed after a period of withdrawal occurs independent of olfaction.

## DISCUSSION

Olfaction is a primary means by which animals discover and recognize external cues for behavioral choices, especially those related to feeding. Odor perception of food evokes autonomic physiological reactions, such as increased flow of saliva and gastric acid (Lee and Linden, 1992; Feldman and Richardson, 1986). Unsurprisingly, loss of olfaction causes changes in metabolism and thermoregulation, and obesity-related metabolic imbalance is deeply correlated with olfactory performance (Riera et al., 2017; Weinstock et al., 1993; Takase et al., 2016; Thiebaud et al., 2014; Richardson et al., 2004).

Interestingly, we find that olfactory cues alone do not sufficiently impact SD consumption regardless of previous short- or long-term HFD accessibility, implying that olfactory cues of inaccessible HFD are not enough to change the perceived value of HFD. Furthermore, general olfactory sensitivity and detection of food are enhanced in the calorically deprived state (Prud’-homme et al., 2009; Aimé et al., 2007; Pager, 1974), although this is debated in humans (Stafford and Welbeck, 2011; Albrecht et al., 2009). Although it is theorized that the ethological purpose of this enhancement is to aid food foraging, we show here that olfactory cues of HFD are not necessary or sufficient to change feeding patterns regardless of hunger state. Additionally, the rigidity of consumption patterns persists beyond novelty of the HFD, suggesting that animals quickly learn the relevance of a food substance based on accessibility and not on olfactory cues. Ingestive or post-ingestive effects are therefore likely more impactful than pre-consumptive sensory cues.

Beyond the relationship between metabolic state and olfactory cues, other consummatory chemosensory experiences may be necessary for prandial choice. Gustatory perception, although decreased, is still present during anosmia (Mozell et al., 1969; Liu et al., 2020). Although numerous receptors and signaling mechanisms have been identified that encode the different types of taste, a global aguesic rodent model is lacking (Yarmolinsky et al., 2009). Interestingly, reduced gustatory perception of sweetness in people with gestational or type 2 diabetes was associated with increased preference for sweet substances (Tepper et al., 1996; Tepper and Seldner, 1999). This preference may partly be influenced by trigeminal sensations, like texture, which is important for maintained preference (Harada et al., 2020; Jeltema et al., 2016; Naim et al., 1986).

How do we reconcile our data with previous research showing that loss of olfaction reduces diet-induced obesity phenotypes? We retain two distinctions in our study. First, we tested our mice after 2–3 weeks of recovery from an irreversible, anosmic surgical procedure to allow an adjustment period to the loss of smell. Removal of olfaction, either temporary or permanent, can result in robust behavioral changes, such as increased depressive-like symptoms, hyperactivity, hyperirritability, and stress (van Riezen et al., 1977; Song and Leonard, 2005; Cairncross et al., 1977). Temporary methods of inducing anosmia are brief, with recovery of olfaction beginning days post-surgery (Harding and Wright, 1979; Kinney et al., 1993). Thus, behavioral testing with transitory methods must be completed soon after surgery or manipulation. This accelerated assessment can often obscure results as it becomes difficult to assign physiological and/or behavioral defects to deficits in smell or the myriad of aversive consequences arising from the acute loss of olfaction. In contrast, because we provided a recovery period to allow for anosmic acclimation after olfactory bulbectomy, we were able to distinguish changes in feeding separate from those induced from surgery.

Second, we tested our mice with an introduction to HFD after anosmia had already occurred. Because nutritional value can be conditioned by taste, texture, caloric density, and/or reward value by post-ingestive signaling (Bolles et al., 1981; Elizalde and Sclafani, 1990), it is possible that animals learn to compensate for the inability to smell post-recovery to target HFD consumption. Further studies are necessary to pinpoint the exact mechanism(s) responsible for HFD preference and SD devaluation. Sham feeding experiments whereby HFD is consumed but not digested or absorbed will help elucidate these physiological processes. The relative contribution of taste and texture could be tested by implanting an esophageal fistula that empties calories before reaching the stomach, and gastric and enteric signals could be determined by fistulas embedded directly in the stomach in parallel with a gastric pylorus cuff to prevent nutrient leaking (Williams et al., 2003). These surgeries are currently challenging in mice but are possible in larger mammals like dogs and rats (Cooke et al., 1972; Magee and Naruse, 1983; Miller and Kessen, 1952).

A separate consideration is that olfactory bulb ablation causes long-term physiological changes separate from the loss of olfactory perception. For example, olfactory bulb removal, but not peripheral ablation of cells in the olfactory epithelium, has been shown to induce hyperirritability and increased predatory aggression (van Riezen et al., 1977; Alberts and Friedman, 1972). Additionally, permanent anosmia can affect a multitude of behaviors outside feeding, such as mating, visual discrimination, and non-olfactory-based learning (Rowe and Edwards, 1972; Phillips, 1970; Wenzel and Salzman, 1968). Given that our data show that all mice exhibit a robust HFD preference and SD devaluation regardless of intact olfaction, it is unlikely that olfactory bulb ablation affects visual and/or textual recognition or learning of the caloric composition, density, or rewarding aspects of HFD. This is further reinforced by the comparable intake of HFD upon initial exposure in both the presence and loss of olfactory function paradigms.

In summary, we show that intact odor perception alone does not affect valuation changes toward food choices. These data support the hypothesis that although olfaction is a primary chemosensory node for detection and recognition, the rapid and enduring valuation of food substrates likely relies on alternative systems such as gustatory, mechanosensory, and post-ingestive signaling.

## STAR★METHODS

### RESOURCE AVAILABILITY

#### Lead contact

Further information and requests for resources should be directed to and will be fulfilled by the lead contact, Michael J. Krashes (michael.krashes@nih.gov).

#### Materials availability

This study did not generate new unique reagents.

#### Data and code availability

The published article includes all datasets generated or analyzed during this study.

### EXPERIMENTAL MODEL AND SUBJECT DETAILS

#### Mice

C57BL/6J male and female mice aged > 8 weeks were given *ad libitum* access to water and standard diet (SD) (Envigo 7017 NIH-31, 14% kCal from fat) unless otherwise stated. Mice were initially group-housed, and upon surgery or the start of home cage feeding experiments, mice were then single-housed (12:12 hour light/dark cycle). All animal protocols were approved by the National Institutes of Health Institutional Animal Care and Use Committee.

### METHOD DETAILS

#### Olfactory Bulb Ablation

Mice were divided into a control (sham) group and a bulbectomized (anosmic) group based on birth date, sex, and weight. To eliminate olfaction, mice in the anosmic group underwent an olfactory bulb ablation surgery. Mice were anesthetized with isoflurane, two holes were drilled into the skull, and suction was used to bilaterally remove the olfactory bulb. Sterile packing material (Pfizer Inc, #00009039605) was used to prevent excessive bleeding. Mice in the sham group underwent a similar surgery in which two holes were drilled into the skull but the olfactory bulb was left intact. After the surgery, mice underwent a 2-week recovery period.

#### Anosmia Screening

A 20-minute buried food test (van Riezen et al., 1977) was used to evaluate olfaction functioning in both anosmic and sham mice after the recovery period. Mice were food-restricted overnight for 16–18 hours before the experiment. A chocolate chip and a neutral metal cap of roughly the same size and weight were hidden in two corners of the home cage bedding. The latency to food discovery was recorded. Any anosmic mice with latencies to food within the 99% confidence interval calculated by the sham mice latencies were excluded from this study. At the end of the study, mice were euthanized, and their brains were examined for remaining olfactory bulb tissue. Anosmic mice with any remaining olfactory bulb tissue were removed from the study.

#### Food and Weight Measurements

The weights of the mice and the food intake of both SD and HFD were recorded each week over the course of the study. Caloric intake was calculated by weighing the amount of SD and HFD eaten and then calculating the number of calories in each gram of food eaten (1-g SD = 3 kcal, 1-g HFD = 5.21 kcal).

#### Inaccessible and Short Accessibility Food Experiments

For inaccessible food experiments, all mice underwent a standard diet (SD) feeding baseline of one week. After the baseline period, mice were separated into groups depending on subsequent experiments. One group of mice (control/SD) were maintained on *ad libitum* access to water and SD. Two groups of mice (SD + HFD) were placed on *ad libitum* access to water, SD, and high fat diet (HFD) (Research Diets D12492, 60% kCal from fat). One group of mice (SD + Inaccessible HFD) were placed on *ad libitum* access to water and SD but were given HFD encased in a Tea Ball (Yinggesi Premium Stainless-Steel Tea Filter), such that mice could not physically access the HFD. One group of mice (SD + Short HFD + Inaccessible HFD) were placed on *ad libitum* access to water and SD, given a one-time accessible exposure to a yoked amount of HFD equal to their individual daily caloric load on the first day after the baseline period, and were also given HFD encased in a Tea Ball. During the withdrawal period, all mice had access to only *ad libitum* water and SD, except for one of the two groups of SD + HFD fed mice that were additionally given HFD encased in a Tea Ball for the duration of the withdrawal period.

#### Anosmia Experiments

For the olfactory ablation experiments, all mice underwent a SD feeding baseline of one week. After the baseline period, sham and anosmic mice were separated into groups depending on subsequent experiments. In one group (control), some sham and anosmic mice were maintained on *ad libitum* access to water and SD. In one group (SD + HFD), some sham and anosmic mice were placed on *ad libitum* access to water, SD, and high fat diet (HFD). During the withdrawal period, all mice had access to *ad libitum* water and SD.

#### Fast-Refeed Tests

During fast-refeed experiments, mice were fasted overnight for 16–18 hours and given access to either SD, both SD and HFD, or SD and inaccessible HFD in a tea ball for one hour between 09:00–11:00.

#### High-Fat Diet Novelty Test

After the initial SD 1-week baseline, sated mice in the SD + HFD group were given access to HFD for 1 hour, after which the number of calories consumed in HFD was recorded.

### QUANTIFICATION AND STATISTICAL ANALYSES

Graphpad Prism 8 was used for graph generation and data analyses. Data are presented as ± SEM n = number of animals. Unpaired t tests, two-way ANOVAs (mixed-model) with multiple comparisons, or Pearson’s correlations were used when appropriate. Post hoc analyses were run with Tukey’s Honestly Significant Difference test or Bonferroni’s Multiple Comparisons tests. The level of significance was set at p < 0.05.

## Supplementary Material

Supplemental Material Only

## Figures and Tables

**KEY RESOURCES TABLE:** 

REAGENT or RESOURCE	SOURCE	IDENTIFIER
Software and Algorithms		
Prism 8.0	GraphPad	https://www.graphpad.com/scientific-software/prism/

## Highlights

- Olfactory HFD perception alone does not alter home-cage feeding or induce SD devaluation
- Prior HFD exposure does not enhance salience of HFD olfactory cues
- Olfaction is not required for home-cage HFD preference or SD devaluation
- Intact olfaction is not necessary for devaluation of SD during periods of hunger
