# Supplementary material for: Examining the role of olfaction in dietary choice
Source: Cell Rep. Author manuscript; Available in PMC 2022 Feb 23. (PMC8864488; doi:10.1016/j.celrep.2021.108755)
Supplement: Supplemental Material Only [file NIHMS1776895-supplement-Supplemental_Material_Only.pdf]

Cell Reports, Volume *34*

Supplemental Information

Examining the role of olfaction in dietary choice

Montana H. Boone, Jing Liang-Guallpa, and Michael J. Krashes

Figure S1

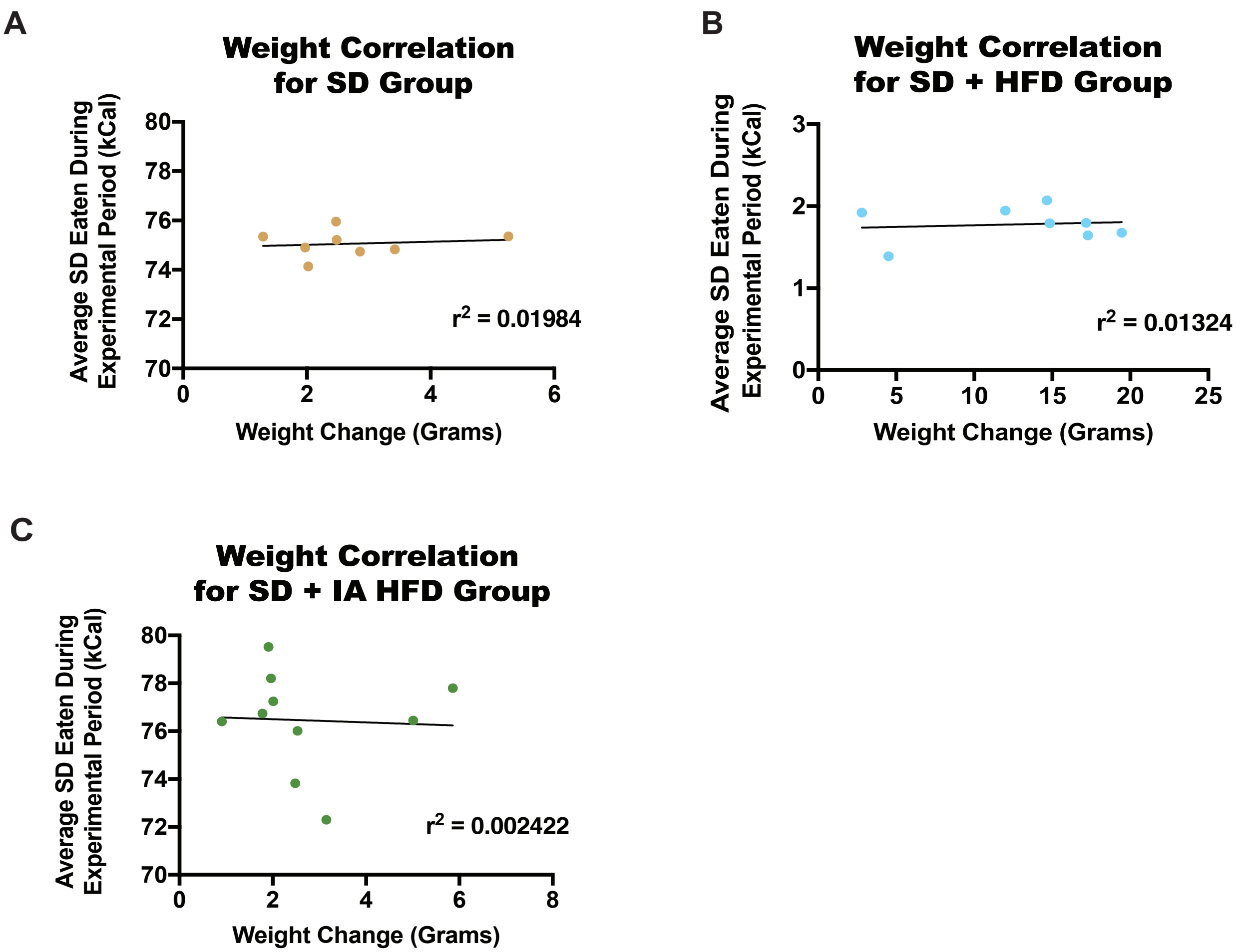
A
Weight Correlation for SD Group
Average SD Eaten During Experimental Period (kCal)
80
78
76
74
72
70
r2 = 0.01984
0
2
4
6
Weight Change (Grams)
B
Weight Correlation for SD + HFD Group
Average SD Eaten During Experimental Period (kCal)
3
2
1
0
r2 = 0.01324
0
5
10
15
20
25
Weight Change (Grams)
C
Weight Correlation for SD + IA HFD Group
Average SD Eaten During Experimental Period (kCal)
80
78
76
74
72
70
r2 = 0.002422
0
2
4
6
8
Weight Change (Grams)

Figure S1: Devaluation of SD is not correlated with weight gain in odor perception studies. Related to Figure 1. No correlation between the average amount of SD intake during the 8 week experimental period and body weight changes in the (A) SD only group, n = 8, Linear regression, R^2=0.01984, P= 0.7394 (B) SD + HFD group, n = 8, Linear regression, R^2=0.01324, P= 0.7861,and (C) SD + Inaccessible HFD Group, n = 9, Linear regression, R^2=0.002422, P= 0.8926. All groups had a mix of males and females. Weight change is calculated as weight at the start of the experimental period subtracted from weight at the end of the experimental period. Average SD eaten is calculated using all weekly SD consumption values during the experimental period.

Figure S2

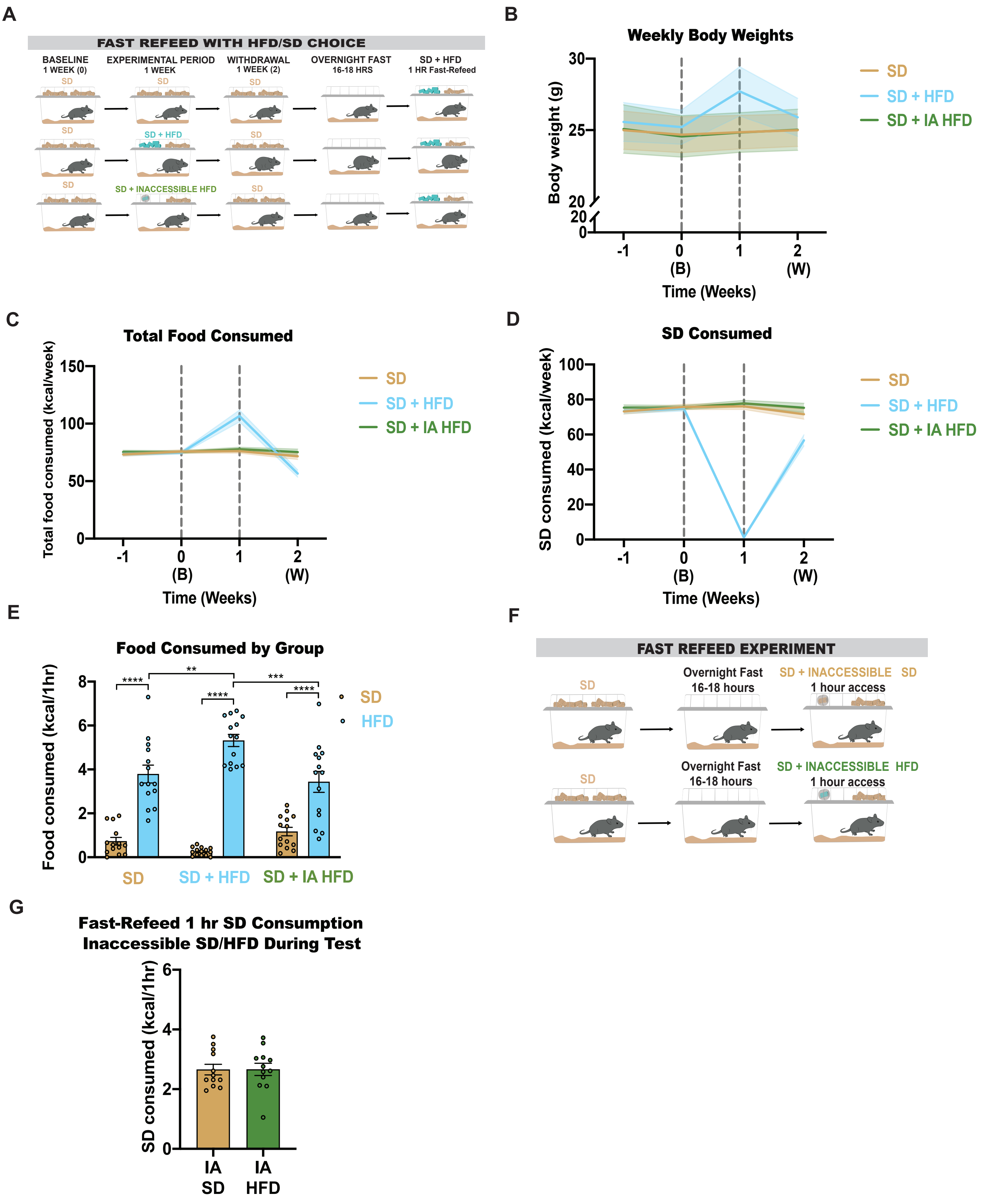
A
FAST REFEED WITH HFD/SD CHOICE
BASELINE
1 WEEK (0)
EXPERIMENTAL PERIOD
1 WEEK
WITHDRAWAL
1 WEEK (2)
OVERNIGHT FAST
16-18 HRS
SD + HFD
1 HR Fast-Refeed
SD
SD + HFD
SD + INACCESSIBLE HFD
B
Weekly Body Weights
Body weight (g)
Time (Weeks)
(B)
(W)
SD
SD + HFD
SD + IA HFD
C
Total Food Consumed
Total food consumed (kcal/week)
Time (Weeks)
D
SD Consumed
SD consumed (kcal/week)
Time (Weeks)
E
Food Consumed by Group
Food consumed (kcal/1hr)
****
**
***
HFD
SD + IA HFD
F
FAST REFEED EXPERIMENT
Overnight Fast
16-18 hours
SD + INACCESSIBLE SD
1 hour access
SD + INACCESSIBLE HFD
G
Fast-Refeed 1 hr SD Consumption
Inaccessible SD/HFD During Test
SD consumed (kcal/1hr)
IA
SD
IA
HFD

Figure S2: Inaccessibility of HFD does not affect feeding to SD or HFD when it becomes accessible. Related to Figure 1. (A) Experimental timeline and group schematic for fast-refeed test with 1 hr SD and HFD access. Weekly measurements of (B) body weight, n = 14 per group (RM two-way ANOVA, Week x Group: $F(6, 117) = 7.212$, $P<0.0001$), (C) total number of calories consumed, n = 14 per group (RM two-way ANOVA, Week x Group: $F(6, 117) = 22.04$, $P<0.0001$), and (D) number of SD calories consumed, n = 14 per group (RM two-way ANOVA, Week x Group: $F(6, 117) = 157.6$, $P<0.0001$) before the SD/HFD choice fast-refeed experiment. (E) Within-subject comparison of 1 hr SD and HFD fast-refeed consumption, n = 14 per group (RM two-way ANOVA, Group x Diet: $F(2, 78) = 11.43$, $P<0.0001$, Tukey's multiple comparisons). (F) Experimental timeline and group schematic for SD fast-refeed test with a novel, inaccessible HFD or familiar, inaccessible SD. (G) Within-subject comparison of 1 hr SD fast-refeed consumption across testing sessions. n = 12 per group (Paired t-test (two-tailed), $P=0.9778$). All groups had a mix of males and females. Dotted lines in **B, C,** and **D** delineate window of HFD availability or inaccessible HFD. B=Baseline. WD=withdrawal. All error bars and shaded regions of **B, C,** and **D** represent mean ± s.e.m. **P<0.01, ***P<0.001, ****P<0.0001.

Figure S3

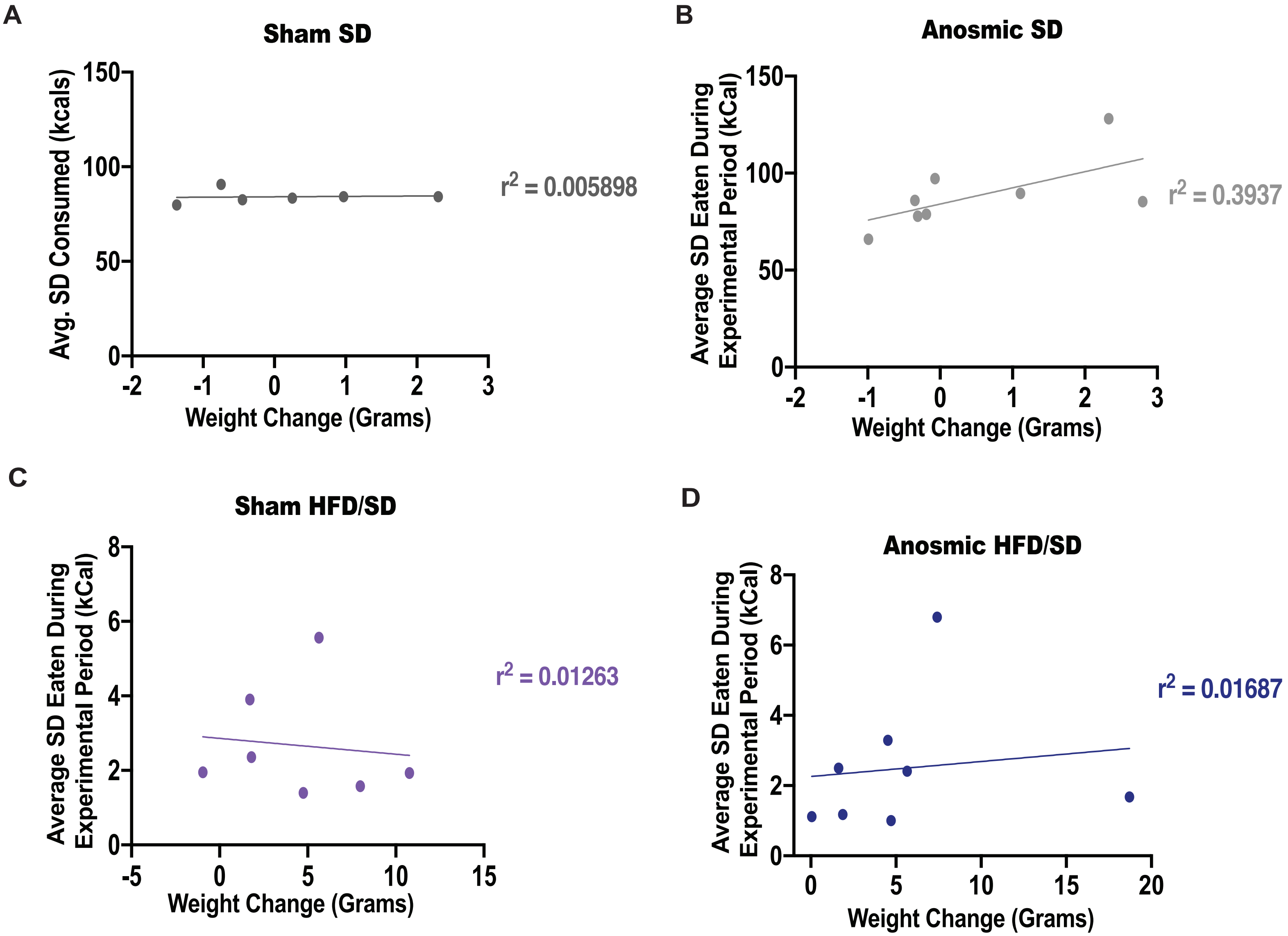
A
Sham SD
Avg. SD Consumed (kcals)
Weight Change (Grams)
r2 = 0.005898
B
Anosmic SD
Average SD Eaten During Experimental Period (kCal)
Weight Change (Grams)
r2 = 0.3937
C
Sham HFD/SD
Average SD Eaten During Experimental Period (kCal)
Weight Change (Grams)
r2 = 0.01263
D
Anosmic HFD/SD
Average SD Eaten During Experimental Period (kCal)
Weight Change (Grams)
r2 = 0.01687

Figure S3: SD devaluation occurs regardless of anosmia and is not correlated with weight gain. Related to Figure 3. (A) sham SD group, n = 6 (Linear regression, R^2=0.005898, P=0.8850), (B) anosmic SD group, n = 8 (Linear regression, R^2=0.3937, P=0.0959), (C) sham HFD + SD group, n = 7 (Linear regression, R^2=0.01263, P=0.8104), and (D) anosmic HFD + SD group, n = 8 (Linear regression, R^2=0.01687, P=0.7592). Weight change is calculated as weight at the start of the experimental period subtracted from weight at the end of the experimental period. Average SD eaten is calculated using all weekly SD consumption values during the experimental period. All groups had a mix of males and females.
